# Indigenous knowledge, use and on-farm management of enset (*Ensete ventricosum* (Welw.) Cheesman) diversity in Wolaita, Southern Ethiopia

**DOI:** 10.1186/1746-4269-10-41

**Published:** 2014-05-08

**Authors:** Temesgen Magule Olango, Bizuayehu Tesfaye, Marcello Catellani, Mario Enrico Pè

**Affiliations:** 1 Institute of Life Sciences, Scuola Superiore Sant’Anna, Piazza Martiri della Libertà 33, 56127 Pisa, Italy; 2 Hawassa University, School of Plant and Horticulture Science, P.O. Box 5, Awassa, Ethiopia

**Keywords:** Enset, Intra-specific diversity, Indigenous knowledge, Landraces, On-farm conservation, Wolaita, Ethiopia

## Abstract

**Background:**

*Ensete ventricosum* (Welw.) Cheesman is a major food security crop in Southern Ethiopia, where it was originally domesticated and during millennia became pivotal crop around which an entire farming system has developed. Although its cultivation is highly localized, the enset-based farming system provides sustenance to more than 20 million people. Precise ethnobotanical information of intra-specific enset diversity and local knowledge on how communities maintain, manage and benefit from enset genetic resources is imperative for the promotion, conservation and improvement of this crop and its farming system.

**Methods:**

This study was conducted in Southern Ethiopia among the Wolaita 'enset culture' community. The research sample consisted of 270 households from 12 *Kebeles* (villages) representing three agro-ecological ranges. By establishing Participatory Rural Appraisal (PRA) based interactions and applying ethnobotanical interviewing methods of free-listing and open-ended questionnaires, information on the use and management of enset diversity, and its associated folk-biosystematics, food traditions and material culture was collected and analyzed.

**Results:**

While enset agriculture is seen as cultural heritage and identity for the Wolaita, enset intra-specific diversity holds scenic, prestige and symbolic values for the household. In the present study we recorded 67 enset landraces under cultivation, and through a comprehensive literature review we identified 28 landraces reported from other areas of Wolaita, but not encountered in our survey. Landraces, identified using 11 descriptors primarily related to agro-morphological traits, are named after perceived places of origin, agro-morphological characteristics and cooking quality attributes. Folk classification of enset is based on its domestication status, 'gender', agro-ecological adaptability and landrace suitability for different food and other uses (fiber, feed, medicinal). Enset as a food crop is used to prepare 10 different dishes in Wolaita, 8 of which are exclusively prepared using enset, and their consumption ranges from daily staple to specialty food in festive occasions and ceremonies. On-farm landrace diversity and richness is guided by household needs; its dynamics is managed through regular propagation, harvesting restrain, control of landrace composition and arrangement in the enset homegardens.

**Conclusions:**

This study reported on the knowledge system, socio-cultural process and community practices that drive the maintenance of intra-specific on-farm enset diversity in Wolaita, Southern Ethiopia. The information is crucial for developing community based complementary *in situ* and *ex situ* conservation strategies to foster conservation of enset genetic resources and associated indigenous knowledge system.

## Introduction

Many crop plants that have been under cultivation for millennia and are contributing to the livelihoods of local communities are scarcely known, if not completely unknown, outside the regions of their cultivations and use. Crops with such localized uses are primarily grown in subsistence farming systems in many Developing Countries, including Ethiopia, and they substantially contribute to food and nutrition security of small-scale farming households [1,2]. *Ensete ventricosum* (Welw.) Cheesman, commonly known as enset, is a monocarpic perennial herb of the Musaceae family originated in Ethiopia [3]. Geographically distributed as a wild species in many parts of Sub-Saharan Africa and Asia [4,5], enset is cultivated only in its native indigenous farming systems of South and South-Western Ethiopia [6]. In fact in Ethiopia, *E. ventricosum* is arguably the most important crop contributing to food security and rural livelihoods for about 1/4 (20 million people) of the Country's population. Enset domestication dates back to Neolithic time or even earlier [6,7], and its farming system is regarded as one of the few ancient and sustainable agricultural systems in Africa [8,9]. Currently, enset cultivation covers over 300,000 ha of land, one of the largest for perennial food crops in the Country [10].

The enset farming system (EFS), where enset is cultivated as perennial plantation in homestead ring in association with other companion crop species growing in main agricultural land, is rich in both inter-specific and intra-specific diversity [11]. The diversity of crop species occurring in EFS resulted from evolutionary processes over the centuries, influenced by environmental variability and domestication processes guided by native cultures, knowledge and traditions. The generation and continued maintenance of on-farm enset diversity is supported by traditional farmers' knowledge and practices. However, recent studies have noted that the enset agricultural system of Ethiopia is changing or has changed in its social, biological and environmental context [11-13]. Nonetheless, enset and its farming system remains scarcely supported by formal researches, and its resources are underutilized as compared to its potential [14]. This limited effort has meant that the potential of enset is underexploited, and its genetic resources and associated indigenous knowledge system (IKS) are put at risk of continuous erosion.

Studies indicate that social attributes of human communities, such as local knowledge, experiences and cultural values, play a substantial role in the sustainable management, conservation and utilization of genetic resources and restoration of agro-ecosystems [15-17]. With the growing interest in conservation of genetic resources in the agro-ecosystem where crop species have evolved (on-farm conservation) [18,19], indigenous knowledge that locals own is important not only for the society owing it, but also for planners, policy makers and scholars for designing conservation and agro-ecosystem restoration strategies.

Indigenous knowledge system (IKS), or otherwise called local knowledge system, is a wide comprehensive concept which includes, but it is not limited to, the botanical knowledge, traditional food knowledge, surrounding environmental and ecological knowledge that enable farming communities to lead stable livelihoods in their environments [20,21]. IKS of a farming community, expressed in the form of folklores and transmitted orally from one generation to next, is embedded in the food cultures, crops planted and animals reared by the farmers, and the environment and ecological setting the community lives in. It is well established that documenting and deploying the local knowledge of farmers' management and use system of agro-biodiversity is a crucial starting point for improving farming systems as well as for fending-off the loss of biocultural diversity [22].

Our study focuses on one of the indigenous 'enset culture' communities of Southern Ethiopia, the Wolaita. The Wolaita are among the ethno-linguistic groups whose agriculture is based on enset, locally known in *Wolaitato Donaa* (the language of the Wolaita) as *uutta*. The Wolaita is regarded as 'the enset people' or 'the people of enset culture' for the strong interlink that exist between enset cultivation and the local food and material culture of the people [23]. However, currently the Wolaita region is among the enset growing zones where landrace diversity and enset culture was reported to be vulnerable to the recent socio-economic and ecological changes occurring in the area [12]. Earlier studies in Wolaita enset farming systems have focused on inventorying landrace diversity, documenting cultivation and processing practices [11,24]. Equally important, but less researched and not systematically documented, is the way the local people maintain, manage and benefit from enset diversity in Wolaita.

The present study was undertaken with the objective of (i) exploring the status and extent of enset cultivation and diversity; (ii) investigating folk-biosystematics of enset landraces; (iii) documenting socio-cultural, dietary, ethno-medicinal and other related uses of enset; and (iv) investigating community practices relevant to maintenance of on-farm landrace diversity in Wolaita.

## Research methods

### Area of the study and local context

**Figure 1:**
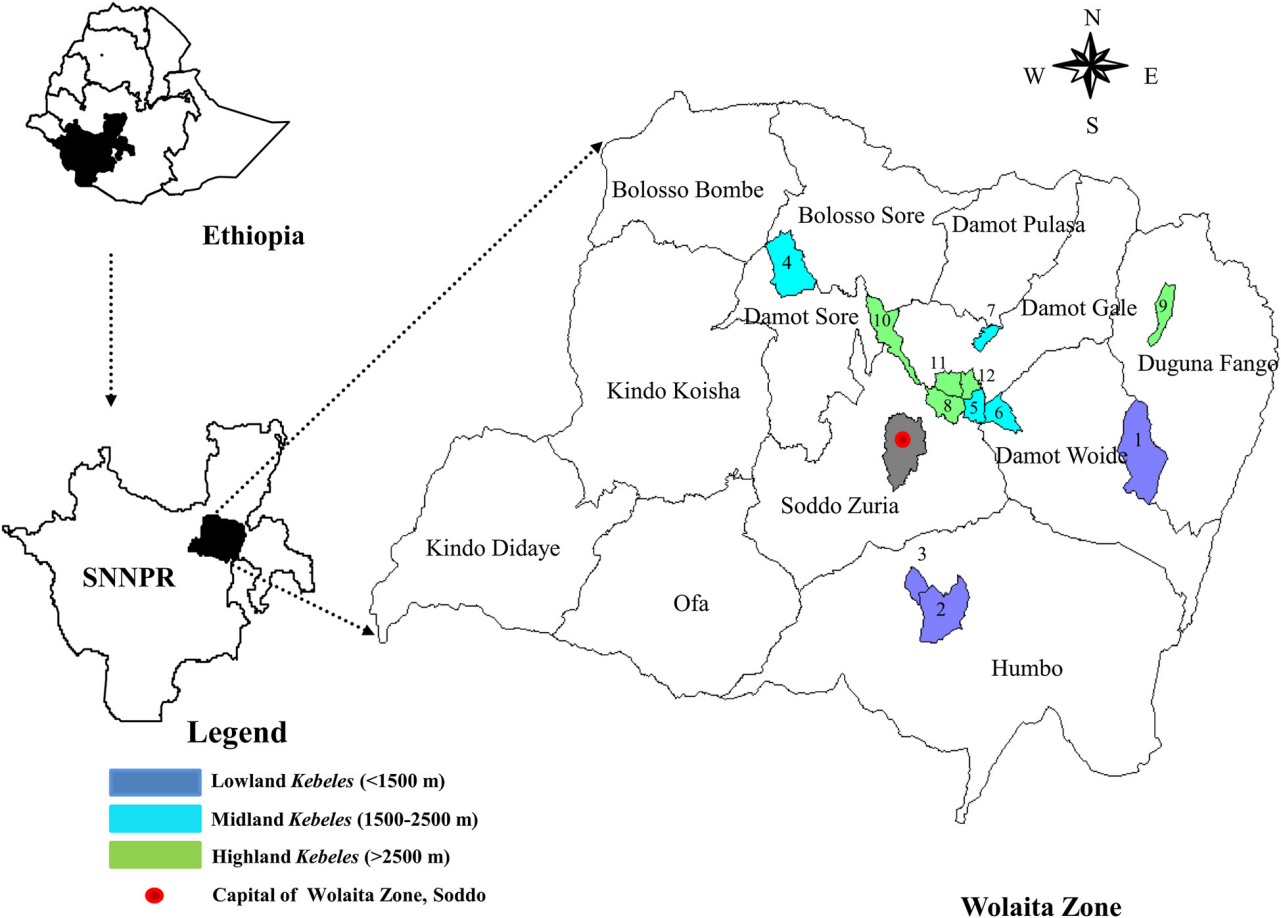
**Location of the *Kebeles *and administrative *Woredas *in Wolaita Zone of Southern Nations Nationalities and Peoples' Region (SNNPR), Ethiopia, object of the present study.** Numbers indicate *Kebeles*: 1 = Anka-shashara; 2 = Ella-kaballa; 3 = Shochora-agodama; 4 = Afama-amino; 5 = Delbo-atwaro; 6 = Mayokote; 7 = Sebaye-korke; 8 = Delbo-wogene; 9 = Dugunaoffa-kalacha; 10 = Gurumo-koysha; 11 = Shasha-gale; 12 = Woshi-gale.

With an area of approximately 438,370 hectares, and an estimated population of 1,750,830, the Wolaita administrative Zone is part of the Southern Nations, Nationalities and Peoples' Region (SNNPR) of Ethiopia; SNNPR is one the 9 Regions and 3 Chartered Cities in which the Federal Democratic Republic of Ethiopia is composed [25]. The Regions are sub-divided into Zones, which are organized into *Woredas*. Within *Woredas*, *Kebeles* are the smallest administrative units. Geographically, Wolaita Zone is located between 7° 00' North latitude and 37° 45' East longitude at the edge of the East African Great Rift Valley. Inhabitants of the Wolaita Zone are primarily the Wolaita ethno-linguistic communities speaking the Omotic Wolaita language, *Wolaitato Donaa*. The Wolaita are predominantly agriculturalists, practicing mixed crop-livestock production and living in permanent settlements. Within their landholdings, community members maintain fruit orchards, nurseries, medicinal plants, vegetables, root and tuber crops, ornamentals, spices, as well as open areas for raising domestic animals [11,26]. Enset cultivation is the centre of the cropping system in which the entire farming system is based and the crop is the major food security and livelihood source [26]. Animal husbandry is also an important economic activity in the area and includes apiculture, poultry, small-ruminants and livestock rearing. Income from crop production and animal husbandry is supplemented by activities such as handicraft (blacksmithing, weaving and pottery) and trade in the area. The present study was conducted in 12 *Kebeles* belonging to 6 administrative *Woredas* in the Wolaita Zone (Figure 1 and Table 1). *Kebeles* were selected on the basis of enset growing potential and agro-ecological (altitude) variation; and the sampled territory covered main enset growing agro-ecological zones (1,500 – 2,800 m above sea level) of Wolaita.

### Sampling procedure

A stratified random sampling procedure was followed to define the sampling unit. Based on the three traditional agro-ecological zones of Ethiopia, lowland <1,500 m, midland 1,500-2,500 m and highland >2,500 m above sea level [27], the enset farming system of Wolaita was stratified in terms of elevation ranges. Since enset is primarily cultivated in the midland and highland regions of Wolaita and other parts of Southern Ethiopia [9], 5 *Kebeles* from highland, 4 *Kebeles* from midland and 3 *Kebeles* from lowland were randomly selected. In the process of *Kebele* selection, key informants comprising agricultural officers and Development Agents (DAs) were consulted. The number of consulted key informants in all *Woredas* was 10; but 9 key informants in Damot Woyide and Soddo Zuria *Woredas* as well as 8 key informants in Duguna Fango *Woreda* were participated. From the 12 selected *Kebeles*, a total of 270 households randomly drown from resident booklets were considered as over all sample size for the study. In 9 of the *Kebeles*, 25 households from each *Kebele* were selected for individual interviews and from each of the remaining 3 *Kebeles* (Sebaye-korke, Shasha-gale and Woshi-gale) 15 households were chosen and interviewed. Table 1 lists the 12 sampled *Kebeles* from 3 agro-ecologies and 6 different *Woredas*. Respective area (km^2^), population size, elevation range and number of households sampled in each of the *Kebeles* are also indicated.

**Table 1:** ***Kebeles *** **under study, their area size, altitude range, resident population size and sample household size**

Study eco-sites (altitude range*)	**Administrative *Woredas***^**‡**^	**Administrative *Kebele***^**‡**^	**Area (Sq. Km)**	**Population**	Altitude range*	**N° of households**
Kolla (lowland) < 1,500 m	Damot Woyide	Anka-shashara	33.62	3,180^§^	1,360-1,460	25
	Humbo	Ella-kaballa	24.48	3,499	1,420-1,640	25
	Humbo	Shochora-agodama	5.47	2,789	1,360-1,640	25
Woina-Dega (Midland) 1,500 -2,500 m	Boloso Sore	Afama-amino	22.34	15,619	1,880-2,140	25
	Soddo Zuria	Delbo-atwaro	4.61	5,650	2,120-2,280	25
	Damot Woyide	Mayokote	8.78	5,579^§^	1,900-2,120	25
	Damot Gale	Sebaye-korke	3.89	2,995	1,960-2,020	15
Dega (Highland) > 2,500 m	Soddo Zuria	Delbo-wogene	5.05	5,949	2,120-2,940	25
	Duguna Fango	Dugunaoffa-kalacha	8.48	4,124^§^	1,800-2,550	25
	Soddo Zuria	Gurumo-koysha	15.23	12,007	1,820-2,780	25
	Damot Gale	Shasha-gale	5.41	3,854	2,100-2,520	15
	Damot Gale	Woshi-gale	6.40	4,607	2,220-2,820	15

*Source*: ZoDARD, Wolaita; ^§^from Woreda Bureau of Agriculture (WBoA) others from SNNPR Livelihood Woreda Reports [28]; ^**‡**^In Ethiopia administration and governance is based on hierarchical units, *Kebele* is the smallest administrative unit and *Woreda* is the next upper unit of administration. Our study covered 12 *Kebeles* from 6 *Woredas*; *traditionally agro-ecologies are categorized into lowland (<1,500 m), midland (1,500-2,500 m) and highland (>2,500 m) above sea level in Ethiopia [27].

### Data collection

Data were collected by combination of methodologies for the acquisition of local knowledge, including literature review, Participatory Rural Appraisal (PRA) tools, focus group discussions (FGD), in-depth individual interviews, expert elicitations and observations of cultivation techniques [29,30].

Literature review provided the necessary background context of enset farming systems and cultural links to the farming communities of Wolaita. Present status of enset agriculture and diversity in Wolaita was reviewed from several published and unpublished sources and reports.

Focus Group Discussions were held in each of the selected *Kebeles* involving members from local administration, community elders, key informant farmer groups and other members of participating communities, and a full consent of collaboration based on the principle of Free Prior Informed Consent was granted [31]. Individual interviews were carried out together with trained enumerators, who are development agents (DAs) working closely with the communities in the respective selected *Kebeles*. Open questions and free-listing approaches were followed to gather information on enset landraces, in particular to assess farmers' perception of landrace diversity, vernacular naming, meaning of names, folk-biosystematics and description of use-values. Food traditions, such as names and preparation of dishes from enset, occasions of consumption and indigenous folklores, such as sayings, poems and songs associated to enset agriculture expressing the cultural tie of the society to the crop, were also documented. Interviews were conducted during drinks and coffee times in homes or homegardens, where the selected household and other interested people were gathered together. Because women of rural Wolaita are particularly responsible from the propagation, protection, harvesting, processing and storage to the final preparation of enset foods [11], they were encouraged to participate to the study and their knowledge, thoughts and opinions were incorporated.

### Data verification and analysis

Collected data were carefully cross-checked for completeness and reliability. Expert elicitations, key informant comments and informal discussion with farmer groups were conducted to verify inconsistencies, enrich and validate information gathered from individual interviews. Descriptive statistical summaries such as frequencies, percentages and averages were calculated using Microsoft Excel 2007 and venn diagrams were made using the online platform VENNY [32].

## Results

### Status and importance of enset cultivation

**Figure 2:**
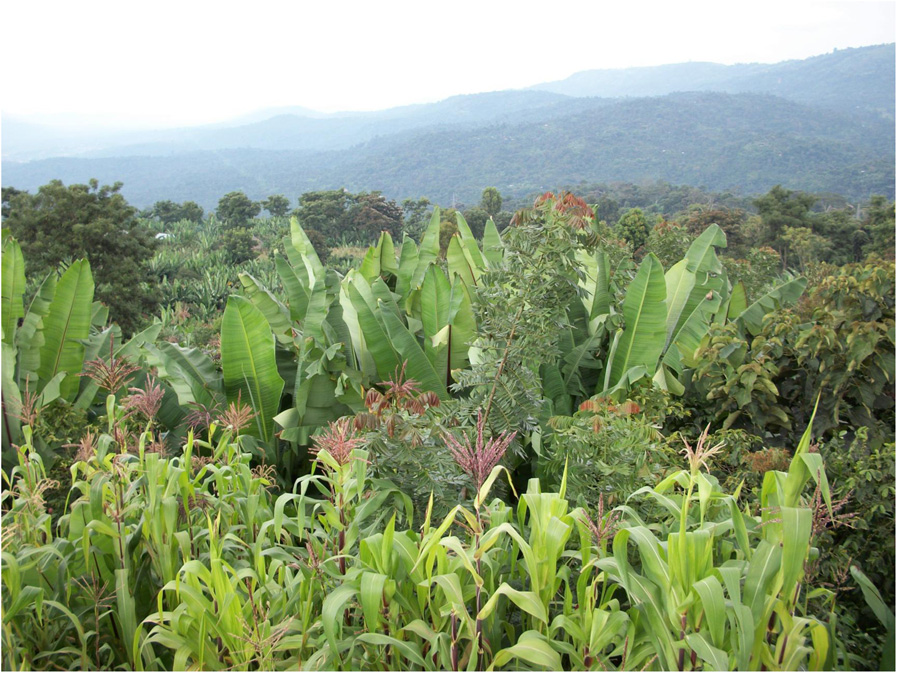
Enset cropping system in Wolaita area. An example of intercropped enset landraces (in the middle) cultivated with coffee (on the right), maize (in front), and other perennial tree species.

Enset cultivation occupies a central position in the agricultural systems of the Wolaita, and every farming household cultivates enset in its homegarden. In the study area, enset is maintained in homegarden (*darkuwa*) ring in poly-varietal perennial plantations without any crop-rotations and land-fallowing. Sometimes, farmers maintain enset landraces intercropped with perennial tree crops, such as coffee (*Coffea arabica* L.), avocado (*Persea americana* Mill.), guava (*Psidium guajava* L.), and annual and biennial crops, such as maize (*Zea mays* L.), Ethiopian kale (*Brassica carinata* A. Braun) and yam (*Dioscorea* spp.) (Figure 2). Enset is one of the most widely cultivated crop in Wolaita, primarily in midland and highland areas, although it covers a relatively smaller area per unit of production compared to cereals and pulses. When asked about the importance of enset, farmers indicated enset as a multi-purpose crop available all year-round, and that needs only household produced inputs for its production. The farming communities and Zone Department of Agricultural and Rural Development (ZoDARD) define enset as the most important crop for livelihoods and food security in the Wolaita Zone. Table 2 summarizes some of the reasons why farming communities consider enset as the most important crop in their livelihoods and agricultural systems.

**Table 2:** Farmers' reasons for considering enset as an important crop in their livelihood and agricultural systems

**Reasons**	**Importance**
● Socio-cultural significance as status symbol	Very important
● High household material culture benefits	Very important
● Flexibility in farming systems as an intercrop with annual and perennial crops	Very important
● Drought tolerance	Very important
● Suitability for preparation of staple and high social values dishes	Very important
● Storability of enset products for long periods	Very important
● Possibility of harvesting at any time of the year	Very important
● Use for integration of crop-livestock system	Very important
● Use for production of high quality fiber	Very important
● Use as water source from pseudostem	Important
● Use as firewood source mainly from dried plant parts	Important
● Generating income from sales of propagules, processed food products and fiber	Important
● Medicinal purposes for humans and livestock (e.g. abortifacient, use for placenta delivery)	Important

### Extent of enset diversity

**Figure 3:**
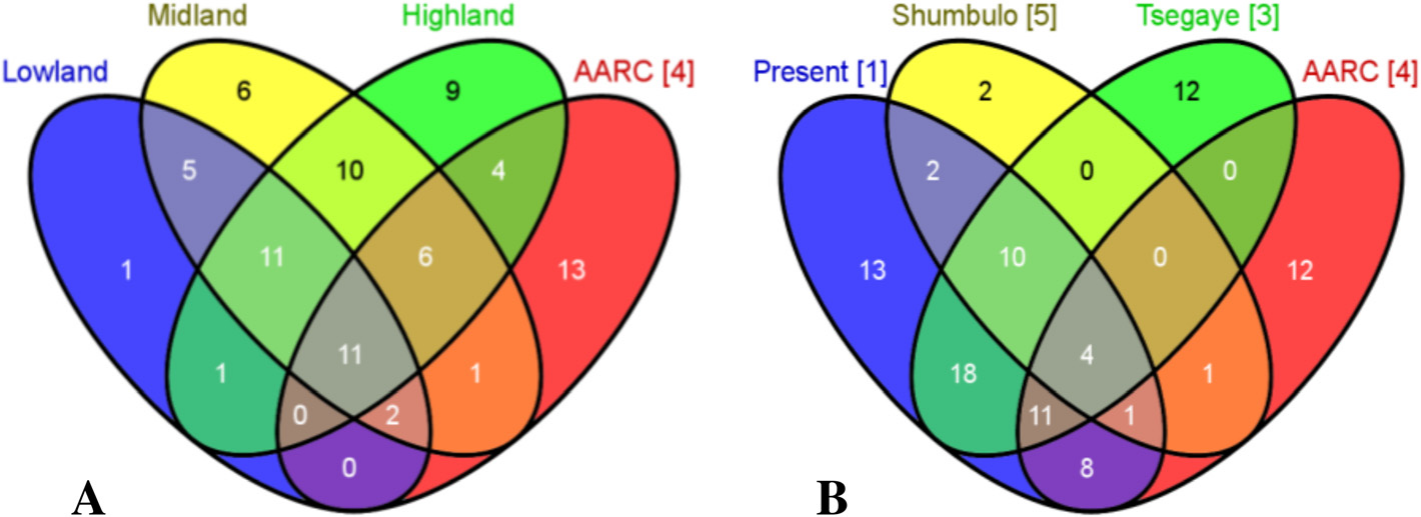
**Enset landraces known to the farming communities of Wolaita in three different agro-ecological zones and *ex situ *collections of AARC (A) and according to different reports and AARC collections (B).** In **A**, traditional agro-ecologies are categorized into lowland (<1,500 m), midland (1,500-2,500 m) and highland (>2,500 m) above sea level in Ethiopia. In **B**, references are (1): the present study; (5): Shumbulo et al., 2012 [24]; (3): Tsegaye and Struik, 2002 [11]; (4): Areka Agricultural Research Centre (AARC) (7° 09' N latitude and 37° 47' E longitudes) maintains national enset landrace collections that also include accessions from Wolaita (Haile, 2014 [33]).

The Wolaita hold a great repository of enset landrace diversity in their homegardens. According to the informants, their agricultural systems maintain a greater level of enset intra-specific diversity than any other crop species. In the present study, 67 different vernacular names of enset landrace under cultivation were recorded. Thirty-one landraces in lowland and 52 landraces in each of the highland and midland agro-ecologies were documented, 22 of which were shared across the 3 agro-ecologies. Unique landrace names that were reported by farmers only in lowland, midland and highland agro-ecologies were 1 (*Bora*), 7 and 13 respectively (Figure 3-A). It is interesting to note that when the recorded landrace names were compared to those of the 37 landraces collected from Wolaita and maintained *ex situ* in the Areka Agricultural Research Centre (AARC), 24 were also identified in the present study. In relation to the agro-ecological distribution of landraces recorded in this study, a total of 21 landraces from highland, 19 from midland and 13 from lowland were also found in the AARC collection, and 11 of those were shared in the three agro-ecologies and AARC (Figure 3-A). We compared the landrace names we recorded with those reported from 5 available previous studies conducted in Wolaita, and with the Wolaita accessions currently maintained in the AARC collection: we identified a total of 95 enset landrace vernacular names known to the Wolaita farming communities (Table 3). Thirteen landraces are unique to the present study, while 4 landraces, *Adinona*, *Godariya, Kekeruwa* and *Tuzuma*, were widely distributed and also present in the AARC *ex situ* collection (Figure 3-B). In general, many landraces as identified by vernacular names, showed a narrow and unique pattern of distribution, whereas 39 (41%) landraces known to the Wolaita community were commonly reported at least by 3 of the 5 studies, including ours [11,24,33,34]. Our analysis indicates that, overall, only 37 (39%) of the landraces that are known to the Wolaita farming communities are represented in the national *ex situ* enset collection of AARC at the time of our survey in 2012. Farmers indicated, both in focus group discussion as well as in individual interviews, that there is a decreasing trend in maintaining landrace diversity in Wolaita. According to the respondents, some of the landraces have been rare; many more are not cultivated anymore.

**Table 3:** **Enset landrace names known to the Wolaita community as reported in the literature, currently maintained in *ex situ *** **collections and recorded in the present study from Wolaita Zone, Southern Ethiopia**

**Vernacular names**	*References	‡**Distribution (study reports)**	**§Distribution (agro-ecology)**	**Vernacular names**	**References**	**Distribution (study reports)**	**Distribution (agro-ecology)**
*Achaka*	[1,3,4]	Medium	M, H	*Hoiya*	[1]	Unique	H
*Adinona*	[1-5]	Cosmopolitan	L,M,H	*Kabaria*	[1-3]	Medium	L,M,H
*Aduwa*	[1]	Unique	L,H	*Kambata*	[1,4]	Narrow	M,H
*Afamma*	[3]	Unique	NI	*Kambata-maziya*	[1]	Unique	H
*Agino*	[1-4]	Common	L,M,H	*Kataniya*	[1-3,5]	Common	L,M,H
*Ala-genna*	[1-3]	Medium	L,M,H	*Kekeruwa*	[1-5]	Cosmopolitan	M,H
*Anko-genna*	[1-4]	Common	L,M,H	*Koltua*	[2]	Unique	NI
*Ankuwa*	[1,4]	Narrow	L,M	*Kuania*	[1,3,4]	Medium	M
*Argama*	[1,3,5]	Medium	M, H	*Kucha-arkiya*	[1,4]	Narrow	H
*Arkiya*	[1-3,5]	Common	L,M,H	*Kuchia*	[1,3]	Narrow	L,M
*Badadia*	[1-4]	Common	L,M,H	*Lalukiya*	[1]	Unique	H
*Bala*	[5]	Unique	NI	*Lembuwa*	[1-3,5]	Common	M,H
*Banga*	[1,3,4]	Medium	M, H	*Locha*	[4]	Unique	NI
*Benuwa*	[1,3]	Narrow	M	*Lochingia*	[1-3]	Medium	M,H
*Bora*	[1]	Unique	L	*Mahia*	[3]	Unique	NI
*Boroda-wanadiyia*	[1]	Unique	H	*Masa-maziya*	[1]	Unique	M,H
*Bota-arkiya*	[1]	Unique	L,M	*Masmasa*	[3]	Unique	NI
*Botya*	[4]	Unique	NI	*Matiya*	[1-4]	Common	L,M,H
*Budaro*	[3]	Unique	NI	*Maziya*	[1-3,5]	Common	L,M,H
*Bukinia*	[1,3]	Narrow	H	*Messa*	[4]	Unique	NI
*Buluwa*	[1,2,4]	Medium	M,H	*Mochiya*	[1-4]	Common	L,M,H
*Bundiya*	[1,3]	Narrow	M	*Nakaka*	[1-3,5]	Common	L,M,H
*Chemia*	[1,4]	Narrow	L,M	*Oso-gurzo*	[4]	Unique	NI
*Chichia*	[1-3,5]	Common	L,M,H	*Peluwa*	[1-3,5]	Common	M,H
*Chorore*	[3]	Unique	NI	*Pena*	[1-3]	Medium	M,H
*Dalulia*	[1-3]	Medium	L,M	*Pokuwa*	[4]	Unique	NI
*Dawro-arkiya*	[1]	Unique	M,H	*Posha*	[4]	Unique	NI
*Dirbuwa*	[1,4]	Narrow	M,H	*Sanka*	[1-3]	Medium	L,M
*Dokozuwa*	[4]	Unique	NI	*Sassa*	[1]	Unique	H
*Dokuwa*	[1-3,5]	Common	M	*Separa*	[3]	Unique	NI
*Erasha*	[4]	Unique	NI	*Shalakumiya*	[1-3]	Medium	L,M,H
*Eslammia*	[1,4]	Narrow	H	*Shamaruwa*	[1-4]	Common	H
*Falakiya*	[1,5]	Narrow	M,H	*Shedodiniya*	[4]	Unique	NI
*Fara*	[1,3]	Narrow	H	*Shuchafe-godariya*	[1]	Unique	H
*Fenku*	[4]	Unique	NI	*Shuchafiya*	[1,5]	Narrow	H
*Gassa*	[1]	Unique	M	*Silqantiya*	[1-3]	Medium	M
*Gefetanuwa*	[1-4]	Common	L,M,H	*Siraria*	[1-3]	Medium	M
*Genaowo*	[1,4]	Narrow	H	*Siskela*	[3]	Unique	NI
*Genessa*	[1-4]	Common	L,M,H	*Sorgiya*	[5]	Unique	NI
*Genna*	[1,2,4,5]	Common	L,M,H	*Suitia*	[1-3]	Medium	L,M,H
*Gezetiya*	[4]	Unique	NI	*Tagacha*	[3]	Unique	NI
*Gishera*	[3]	Unique	NI	*Tenna*	[3]	Unique	NI
*Godariya*	[1-5]	Cosmopolitan	L,M,H	*Tuffa*	[1]	Unique	L,M
*Gomorcha*	[4]	Unique	NI	*Tuzuma*	[1-5]	Cosmopolitan	L,M,H
*Gonwassa*	[1,3]	Narrow	M,H	*Wanadiyia*	[1-3]	Medium	L,M,H
*Guniashia*	[3]	Unique	NI	*Woisha*	[4,5]	Narrow	NI
*Halla*	[1-3,5]	Common	L,M,H	*Zinkiya*	[1-3]	Medium	M,H
*Hawsakuwa*	[3]	Unique	NI				

*References are (1): the present study; (2): Eyasu, 2003 [34]; (3): Tsegaye and Struik, 2002 [11]; (4): Haile, 2014 [33] (Landraces from Areka Agricultural Research Centre (AARC) (7° 09' N latitude and 37° 47' E longitudes) which maintains national enset landrace collections that also include collections from Wolaita); (5): Shumbulo et al., 2012 [24] (which reports identification of 55 landraces but only reported 20 landrace vernacular names).

‡Distribution refers to landrace vernacular names in five of the studies (the present study, Eyasu, 2003 [34], Tsegaye and Struik, 2002 [11], AARC, Haile, 2014 [33] and Shumbulo et al., 2012 [24]); landrace name/s reported only in 1 study = Unique; 2 studies = Narrow; 3 studies = Medium; 4 studies = Common; and 5 studies = Cosmopolitan.

§Distribution across traditional agro-ecologies in Ethiopia viz: lowland (L) (<1,500 m); midland (M) (1,500-2,500 m) and highland (H) (>2,500 m) above sea level. NI= Not Indicated.

### Indigenous knowledge systems of enset diversity

The farmers of Wolaita have a profound knowledge about their enset crop, its diversity and farming system. The specific IKS the community has developed over the years through empirical observation and interaction with enset and its farming system is manifested through (i) the folk biosystematics used for intra-specific enset diversity, (ii) the complex and overlapping uses derived from enset landraces, and (iii) the dynamic on-farm management practices followed to maintain landrace diversity. All the respondents believe that they have inherited this IKS from their forefathers and the custom sustained since time immemorial.

### Indigenous biosystematics: identification, naming and classification

The local farmers of Wolaita we approached perceive each enset landrace they grow as distinct, with clearly distinguishable peculiar characteristics. The farmers use three folk processes of indigenous biosystematics for their landrace under cultivation: they identify the landraces and then name and classify them ***.***

#### Identification of enset landraces

For identification, local farmers used 11 descriptors (Table 4). Those descriptors are related to: morphological characteristics (pseudostem color, midrib color, petiole patches/strips colors), agronomic characteristics (reaction to drought, reaction to disease and pests, maturity time). If in doubt, farmers also use sap color, corm shape and corm color for identification. The local farmers use combinations of descriptors and when asked for key identification characteristics, they referred first to the morphological characters of a landrace. Character descriptors related to the use-value (uses for food, fiber, fodder, medicinal), culinary quality and agronomic characteristics came only after morphological characteristics. Depending on the landraces cultivated in the homegardens, the most frequently mentioned descriptors for identification were leaf color (84% of the respondents), plant size (83% of the respondents) and pseudostem color (82% of the respondents). Some descriptors (sap color and corm color) were used for the identification of a limited number of landraces. Sap color as descriptor was used specifically for landrace *Suitia*, which means 'the bleeding', referring to the red sap color of *Suitia* as compared to the watery and milky sap color of most of the other landraces. Sap color and corm characteristics, mentioned by 57% and 40% of the respondents, were less frequently quoted descriptors for the identification of enset landraces in the study area.

**Table 4:** Farmers' descriptors of enset landraces in Wolaita, Southern Ethiopia

**Identification and characterization criteria**	**Criterion category**	**Examples of representative landraces**	*Respondents (%)
***Plant morphology***			
Pseudostem color	Green	*Achaka, Adinona, Ala-genna, Banga, Lalukiya*	82
	Red	*Kambata, Maziya, Wanadiyia*	
	Dark-purple	*Gefetanuwa, Lochingia, Zinkiya*	
	White	*Agino, Bota-arkiya*	
Leaf color	Green leaves	*Achaka, Adinona, Badadia, Arkiya, Lembuwa*	84
	Purple leaves	*Lochingia*	
Leaf shape and pattern	Narrow erect	*Gefetanuwa, Genessa*	66
	Wide and dropping	*Lembuwa*	
Midrib dorsal color	Red/yellow	*Badadia, Falakiya, Godariya, Eslammia, Maziya*	79
	Purple	*Gefetanuwa, Lochingia*	
	Green	*Ala-genna, Kataniya, Mochiya, Suitia*	
Petiole blotch and patch color	Black	*Ala-genna, Halla, Kabaria, Mochiya, Nakaka*	77
	Brown	*Achaka, Tuzuma*	
^a^Sap color	Red	*Suitia*	57
^a^Corm color	Dark blue strips	*Kabaria, Nakaka, Peluwa*	40
***Plant cycle***			
Maturity	Early	*Gefetanuwa*	66
	Late	*Adinona, Anko-genna*	
***Plant vigor***			
Plant size/height	Vigorous	*Ala-genna, Anko-genna, Godariya, Maziya*	83
	Tiny	*Gefetanuwa, Lochingia, Silqantiya*	
^**b**^ ***Plant reaction to biotic factor***			
Disease and pest	Resistant	*Argama, Halla, Wanadiyia*	62
	Susceptible	*Chichia, Kabaria, Nakaka, Suitia*	
***Plant reaction to abiotic factor***			
Drought	Resistant	*Badadia*, *Gonwassa*	67
	Susceptible	*Kataniya, Nakaka*	

*Quotes are percentages of farmers using identification criteria for distinguishing enset landraces (depending on landrace cultivated in households homegarden).

^a^Sap and corm color are used as identification criteria when farmers are in doubt, hence for conformation.

^b^Disease reaction is specifically considered for bacterial wilt disease (*Xanthomonas campestris* pv. *musacearum*) which is common in the area.

#### Names and naming of enset landraces

Nomenclature is the second folk process the Wolaita employ for their enset landraces after identification. Local farmers in the investigated study area give separate vernacular name for each landrace they grow. The names are often descriptive and reflect variations of landraces in places of origin, morphology, as well as agronomic and cooking characteristics. Often farmers attach names of places in neighboring Zones and *Woredas* to the landrace names (e.g. *Dawro-arkiya, Kucha-arkiya, Kambata, Kambata-maziya*). The naming may include the indication of physical entities (e.g. *Agino*, the Moon), cultivated plants (e.g. *Banga,* barley); domestic animals (e.g. *Bora,* ox, and *Fara,* horse) and of wild animals (e.g. *Godariya*, hyena and *Genessa*, antelope). In other instances, farmers literally used words in *Wolaitato Donaa* to describe the specific morphological, agronomic, and cooking quality attributes of specific landraces (e.g. *Dirbuwa,* fast/hurry, referring to early maturity of the landrace). Table 5 summarizes the names and naming of enset landraces in Wolaita, with their English translation and implied meanings. Most of the landrace names are a single expressions, 'semantically unitary', but 9 of the identified landrace names are structured to 'secondary' names by adding modifier which further describes the landrace (Table 6). For example, landraces names *Bota-arkiya, Dawro-arkiya* and *Kucha-arkiya* are derived from 'primary' landrace name *Arkiya* and the additional modifiers describe color and origin of the *Arkiya* landrace. It should be considered however that, the majority of enset landrace names had their implied meanings unknown to the community members.

**Table 5:** Enset landrace nomenclature after names of places, animals, morphological traits and use-values in Wolaita, Southern Ethiopia

**Naming of landraces**	**Representative landrace vernacular names with their English translation**	**Implication of the vernacular names**
After names of places	*Boroda-wanadiyia* (from Boroda *Woreda*)*, Dawro-arkiya,* (from Dawro Zone)*, Kambata* (from Kambata Zone)*, Kucha-arkiya* (from Kucha *Woreda*)	Origin of the landraces from bordering and nearby *Woredas* and Zones
After names of animals	*Bora* (Oxen- for feeding preference), *Fara* (Horse-feeding preference)*, Genessa* (Antelope-indicating erect leaves like antelopes ear)*, Godariya* (Hyena-to indicate big size and ugly color of the landrace)	*Implying landrace size, color, leave shape, suitability of landrace to feed different animals
After names of crop plants	*Banga* (*Hordeum vulgare* L.),	*Implies taste similarities of the corm of landrace as barley foods
After typical agronomic characteristics	*Dirbuwa* (fast/hurry)	Early maturity of the landrace
After typical morphological characteristics	*Lembuwa* (Dropping), *Lochingia* (Thin),	Leaf Morphology
	*Maziya* (Big, fat) *Silqantiya* (Weak, unable to stand)	Implying plant size and pseudostem strength
	*Agino* (the Moon, related to the Moon's color)	Referring to white/creamy color of pseudostem
	*Suitia* (blood or bleeding, red sap color)	Referring to the sap color
After sensory cooking qualities of landraces	*Chemia* (Bitter not sweet), *Sassa* (silent, not delicious), *Gassa* (offensive bad smell),	Implying landrace food taste and smell
After typical use of landrace	*Tuffa* (fiber, fibrous pseudostems)	Referring to the fibrous nature of the landrace and its importance for fiber
	*Argama* (Clan group in Wolaita)	*Refereeing to the Clan cultivating the landrace
Names given after different physical entities, biological organisms and names of people	*Ankuwa* (Vulcher); *Buluwa* (*Solanum campylacanthum* Hochst. ex A. Rich.); *Mahia* (Tiger) *Sanka* (Door); *Bala* and *Erasha* (Name of a person)	^‡^Unexplained meaning
Difficult to know landrace nomenclatures	*Achaka, Adinona, Ala-genna Lalukiya, Mochiya, Shalakumiya, Shamaruwa* and many others in Table 3	^‡^Unexplained meaning and implication

*Although farmers agree/know the meaning of landrace names they are not sure of its implied meaning; ^‡^The nomenclature and implied meaning wasn't known from the discussions and interviews with the community.

**Table 6:** Sub-variety nomenclature of enset landraces in Wolaita, Southern Ethiopia

**Landrace variety level nomenclature**	**Landrace sub-variety level nomenclature**	**Meanings and implications of sub-variety level names**
*Arkiya*	*Bota-arkiya,*	Color of pseudostem (*Bota*, white)
	*Dawro-arkiya, Kucha-arkiya,*	Names of a place bordering Wolaita ( *Dawro*, *Kucha*)
*Genna*	*Ala-genna, Anko-genna*	*Unexplained meaning
*Godariya*	*Shuchafe-godariya*	*Unexplained meaning
*Maziya*	*Masa-maziya,*	*Unexplained meaning
	*Kambata-maziya*	Name of a place bordering Wolaita
*Wanadiyia*	*Boroda-wanadiyia*	Name of a place but not directly bordering Wolaita

*The 'secondary' nomenclature and implied meaning was not known from the discussions and interviews with the community.

#### Classification of enset landraces

The Wolaita use folk classification systems for their enset landraces, in which different categories of classification overlap. Four criteria are used in classifying enset landrace: (i) domestication status (ii) gender (iii) use-value and (iv) eco-geographic adaptability (Table 7).

**Table 7:** Folk classification of enset landraces in Wolaita, Southern Ethiopia

**Folk classification bases**	Categories*	**Characteristics of landrace in each category**	**Landraces recorded in each category (%) (N = 67)**
Domestication status	Wild *(Talahe uutta)*	Sexually reproduced; occurring naturally in river banks and swampy areas;	NA
	Cultivated	Vegetatively propagated: it occurs in homegarden under farmers' management	67 (100%)
'Gender' of landrace	Female *(Macca uutta)*	Early maturing, more tender, with edible corms	24 (36%)
	Male *(Attuma uutta)*	Late maturing, fibrous, vigorous, stress tolerant, with non-edible corms	28 (42%)
Use-value of landrace	Food use *(Katta uutta)*	Mainly used for enset based foods (*Uncca*, *Itima* and *Doyisa uutta*)	59 (88%)
	Non-food Use *(Harra goa uutta)*	Mainly used as a source for fiber, fodder, firewood, medicine, and water	8 (12%)
Eco-geographic (Altitude) adaptability	Highland *(Geziya uutta)*	Some landraces are preferentially (e.g. *Argama*, *Maziya*) cultivated in highland, although their cultivation also occurs in lowland, otherwise no difference exists between landraces on basis of agro-ecology	NSL
	Lowland *(Gara uutta)*		NSL

*Phrases in parenthesis are in the Wolaita language, *Wolaitato Donaa.*

NA- Not available.

NSL- No specific landrace grown only in highland or lowland was reported.

Farmers distinguish two enset types on the basis of their domestication status, wild or cultivated. Wild enset landraces in Wolaita occur naturally in river banks, swaps, gullies and near streams in highlands (*Geziya*). The Wolaita give the same name for all wild enset, i.e. *talahe uutta*, which means 'the enset of the devil'. Communities of the investigated *Kebeles* believe that there are farmers adding '*talahe uutta*' to cultivation, but there was no farmer encountered in our interview practicing addition of wild enset into cultivation. In the focus group discussion and expert elicitation sessions, participants indicated that it is very unlikely to find wild enset in Wolaita at present.

**Figure 4:**
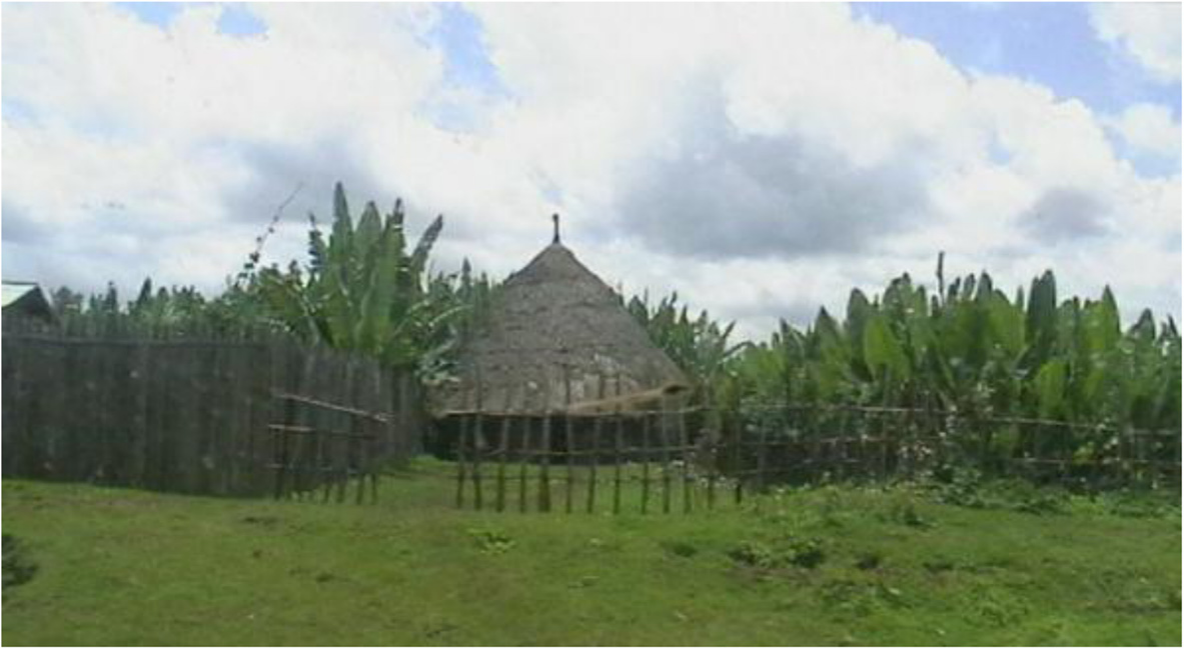
Poly-varietal perennial enset plantations in a homegarden in Wolaita area (Picture kindly provided by Sadik Muzemil, AARC).

Cultivated enset are located in human settlements near dwellings (*darkuwa*) as a homegarden crop (Figure 4). Cultivated landraces are distinguished further by their 'gender', use-values and eco-geographic adaptability (altitude). Farmers classified enset landraces into two major sex categories: 'female' enset (*macca uutta*) and 'male' enset (*attuma uutta)*. The distinction as 'male' and 'female' is not related to the biological reproduction of the landraces. Farmers consider early maturing landraces with high edible corm quality (less fibrous and tender corm), with thin and weak pseudostems, as *macca uutta*, and late maturing fibrous landraces, with corms of poor cooking qualities, as *attuma uutta*. Of the total 67 landraces identified, 24 (36%) were classified as 'female', 28 (42%) as 'male' and the remaining 15 landrace had ambiguous sex designation, some farmers claiming them 'male' and others claiming them 'female'. Based on indigenous use-value, farmers classified enset landraces in two comprehensive use groups: food use and non-food uses (fiber, fodder, firewood, medicinal, construction and water source). Although all enset landraces can be used both for food and non-food uses, there are preferences for specific landrace among communities for particular purposes. In the study area the majority of landraces were primarily planted for food uses and others for non-food use, such as complementing livestock feed, and animal and human medicinal requirements. Very few, such as *Tuffa* and *Lalukiya,* were grown for fiber production and water fetching from their pseudostems respectively. The other classification criterion farmers used was eco-geographical adaptability of landraces. The Wolaita farmers describe major enset ecosystems by elevation regimes: *Gara* (low altitude) and *Geziya* (high altitude); they assign a set of enset landraces to specific elevation niches, i.e. as *Gara uutta and Geziya uutta*. Although landraces adapted to highlands can be cultivated in lowlands and *vice versa*, the growers claim that some landraces (e.g. *Maziya*) are specifically adapted to *Geziya* ecosystems.

### Indigenous uses of enset

#### Cultural identity crop

The enset plant and its cultivation has special cultural meaning and value for the Wolaita. The cultivation of enset also bears a cultural symbol for the communities and it is an expression of their identity. There is a common refrain in the area: '*Nuni uuttan yelletidi uuttan diccida, uutta assa*', (*We were born and grown up on enset*, *we are the people of enset*). According to the information we obtained from the growers, enset is an old crop that has been under cultivation since time immemorial. The cultivation of enset is seen as family heirloom that the antiquity of enset agriculture in their area is what they have heard from their forefathers. The majority (87%) of the farming households surveyed started farming and growing enset crop on the plot of land with a set of landraces inherited from their fathers at adulthood. Locals believe that offering a plot of land with a mixture landraces to an adult son is an old tradition transferred from one generation to next in Wolaita.

Every farming household in Wolaita grows some enset and maintains two or more landraces in its homegarden. Enset being highly valued as a status symbol, people appreciate a hamlet by enset around it and give special respect and name for a man (*Allawa*) owing a large number of vigorously growing enset plants (*Alla)* in his homegarden*.* The Wolaita indicate the cultural uses of enset, its status symbol role and the strong tie the crop has with their life from birth to death using their traditional folklores. Those folklores (see Table 8 for a few examples we were informed of during this study) are quite informative: some of the farmers referred to sayings that compare the difference of knowledge about enset farming between the old and the young and imply the old as expert in the management and knowledge of enset agriculture. Some sayings indicate the status symbol and social position of the owner of a well managed enset homegarden rich in landraces diversity (*O na'i hage giyoi mayuwana, O ketay hage giyoi uttana*), English translation: well dressed kids give prestige to their parents, well managed enset homegardens give prestige to household and hamlet.

**Table 8:** Traditional folklores associated to enset agriculture in Wolaita, Southern Ethiopia

**Sayings in Wolaita language**	**English translations**	**Attributes appreciated and/or implied meanings**
*Uuttane mata dabuwa ixxi eretena metuan gakiyaga*	You don't disagree relatives and enset, who accompany you in bad times	Appreciation of the social value enset has in the community
*Oliyosi bayina uutta, uttiyosi bayina shafa*	No waste from enset, no rest for a river	Appreciation of multipurpose value of all enset plant parts
*Aawai uutta tokidi gatiyosa na'yi banga zeridi gattena*	A land patch a father covers by planting enset cannot be covered by his son by broadcasting barley	Indicating old and new generation differences in cultivation and management of enset (the old are knowledgeable)
*O na'i hage giyoi mayuwana, O ketay hage giyoi uttana*	Well dressed kids give prestige to their parents, well managed enset homegardens (Alla) give prestige to household and hamlet	Indication of enset as symbol of status and prestige crop in the community
*Ika uutte ika guutte?!*	The enset, the tidbit?! (questioning small meal size of enset food served for the hungry who is expecting big meal)	An idiom that implies the notion that enset is a cheap poor-man's food and enough of it should be served generously

#### Staple and cultural food crop

**Figure 5:**
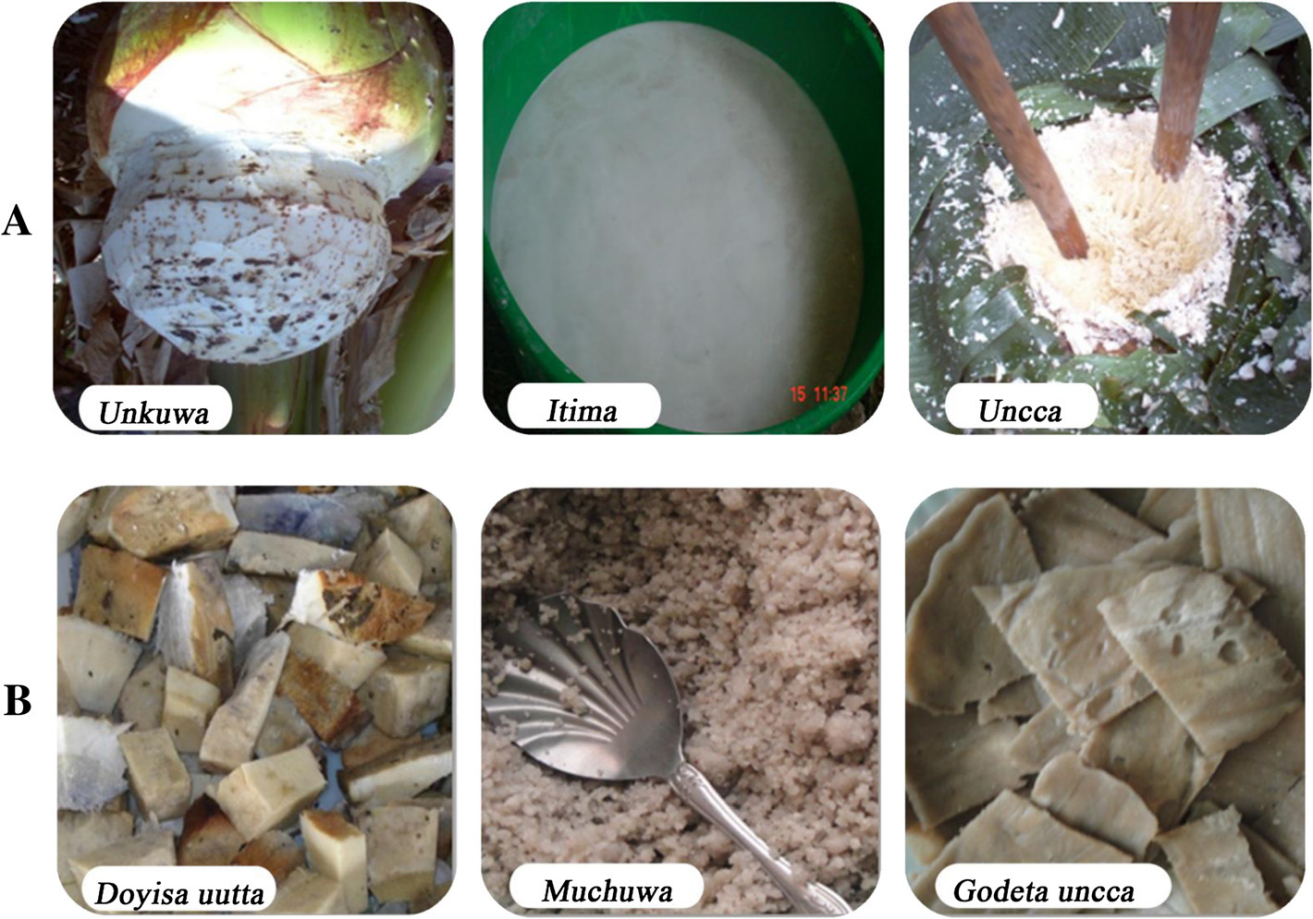
**Examples of enset products and commonly prepared dishes.** In panel **A** three enset primary products are shown. Below each primary product (in panel **B**) one example of a dish that can be prepared from the respective primary product is depicted. (The source of *Itima* and *Doyisa uutta* picture is: Sadik Muzemil, AARC).

Enset foods are served both as staple daily diet as well as in occasions of cultural festivals, hence enset foods have both nutritional and cultural values for the society. In this study, we identified 10 recipes of dishes derived from 3 (*Uncca*, *Itima* and *Unkuwa*) primary enset products (Table 9, Figure 5). *Uncca* and *Itima*, are obtained after processing, whereas the *Unkuwa* or corm is a cooking type boiled and eaten directly without any processing. *Uncca* is a fermented pulp of the enset pseudostem derived by scraping the individual pieces and excluding the fibrous remains, whereas *Itima* is the small amount of water-insoluble starchy product that may be separated from *Uncca* during the processing phase by squeezing and decanting the liquid. Some of the recipes from primary products, such as *Muchuwa* and *Baccira*, are considered a specialty food and served during specific festive occasions. Other recipes, such as *Kintahuwa* and *Saretta*, are considered as 'poor-man's food, often eaten in seasons of food shortages. The growers distinguish preferred enset landraces for different dishes. 'Female' landraces are preferred for *Doyisa uutta* (cooking type), whereas 'male' landraces are preferred for processing. Landraces producing whiter *Itima* after processing are preferred for visually attractive and specialty dishes, such as *Muchuwa*, *Baccira* and *Shendera*.

**Table 9:** The cultural enset foods and recipes of Wolaita, Southern Ethiopia

**Primary enset products**^**‡**^	**Food items**	**Processing and preparation descriptions**	**Use remarks**
*Uncca* (*Kocho*)	*Gola Uncca*	A sort of bread prepared from chopped *Uncca* which has no *Itima*	Fibrous and darker in color relative to *Godeta Uncca*
	*Godeta Uncca*	A sort of bread prepared from an *Uncca*, which is processed from mixtures of *Itima* and inner most leaf-sheath of the pseudostem	Higher quality *Uncca* relative to *Gola Uncca*
	*Kintahuwa*	A sort of bread prepared from inferior quality *Uncca* (i.e. not fully fermented) mainly eaten in seasons of food shortage	Considered as poor-man’s food
	*Bilanduwa*	Prepared from chopped, sieved and roasted *Uncca* by mixing with bean, cabbage, spices and butter	Served as regular staple meal
	*Saretta*	Is a sort of bread prepared from blend of lower quality *Uncca* mixed with maize flour and backed	Considered as poor-man’s food
*Itima* (*Bula*)	*Muchuwa*	Prepared form lightly roasted and cooked *Itima* mixed with butter and spices	Considered as specialty-dish
	*Baccira*	Prepared from *Itima* by mixing it with milk, butter and spices; it is wetter than *Muchuwa*	Eaten on the eve of *Gifaata*, the Wolaita New Year
	Eretta*	Is a porridge prepared from *Itima*, mixed with butter, especially prepared for a postnatal mother (for *Kaacca*)	Considered of high medicinal and nutritional value
	Shendera*	A sort of porridge prepared from *Itima* mixed with butter, but unlike *Eretta* it is commonly eaten by any family member	Prepared for any family members or guests
*Unkuwa* (*Amicho*)	*Doyisa uutta*	Prepared by boiling underground corm (*Unkuwa*), often served with other root crops and vegetables	'*Female landraces*' are preferred

^‡^Primary products are given in Wolaita language (*Wolaitato Donaa*) and in parenthesis *Amharic* (National language of Ethiopia); *those two foods can be prepared from maize and barley, but the rest are specific for enset.

The community indicate that enset foods are traditionally incorporated into cultural events, such as births, deaths, weddings and festivals. During births, a postnatal mother eats *Eretta*, which is a special kind of porridge prepared from *Itima* by mixing it with butter and spices*.* In funeral days, relatives bring *Uncca* (locally termed as *Cana-uncca*), a processed product from enset with extended storability, and mourn together with the family of the deceased. The traditional festival of New Year in Wolaita called *Gifaata* (in September*, Masqala*), is celebrated eating the special foods *Baccira* and *Muchuwa* on the eve and throughout the celebration weeks.

#### Multipurpose material culture crop

The Wolaita do not waste any part of the enset plant. Every part of enset plant has some sort of use in material culture of the Wolaita. The green enset leaf, *yecha*, is used as traditional plate to serve food, or as wrapping material for different products and baking breads, but it can be used also as an umbrella during the rainy seasons. Dried leaves and other pseudostem parts are also collected for making traditional seats (*shill'a*) and mattress (*konashia hitta*). The outer-most dried leaf-sheaths of the pseudostem (*goba*), often harvested while the tree is still growing, is used as a seal (*gossa*) of traditional milk processing pot and for handling butter. Any dried part of the enset plant, mostly the dried lamina (*gombila*) and petiole, is used as a firewood. *Susa*, the dried and semi-dried long lamina, is used for fastening harvests of grasses, crops, and firewood. For construction of fences and traditional houses (*Wolaita ketta*) only dried, water-soaked and relatively strong lamina is used. The fiber of enset, *gola*, produced as side-product during food processing (*Uncca*), is a very strong and high quality fiber. It is used for making ropes, strings, baskets, sacks, house floor carpets, filters, utensils' cleansers and many traditional house decors. Also, the fiber is used for fencing and construction of houses. During our survey, all the interviewed households mentioned one or more of the aforementioned material culture values of enset.

#### Medicinal plant

The enset plant and its parts contribute to indigenous ethno-medicinal values of the Wolaita. Although all the respondents in the study area know and believe that enset is medicinally important, only a few people use it for medicinal purpose. Traditional healers in the area confidentially keep ethno- medicinal knowledge of enset landraces and many other medicinal plant and animal species. Mostly administered in the form of food products, traditional enset medicines include (i) porridge made of *Itima* from *Agino* and *Gefetanuwa* landraces, for strengthening women after delivery, and healing bone fractures in humans respectively; (ii) very highly fermented *Uncca* from *Maziya* and *Halla* landraces, for curing stomach cramps; and (iii) boiled corm of *Lochingia*, for birth control and abortion in humans, and to feed cows to facilitate placental expulsion.

#### Other uses

As described to us by the community, enset agriculture fulfils an ecological role, because it is an organic farming systems using only farmyard manures, with no external chemical fertilizers, herbicides and insecticides. In the study area, animal husbandry is commonly practiced and it benefits from enset agriculture through a year-round supply of nutritious feed; in this regard enset is considered a safety-shield cattle feed because it is available during the drought prevalent seasons of the year, when most other feed sources dry out. The enset plantation in homegardens serves also as a wind break for enset and other crop nurseries. Enset farming also fulfills an aesthetic requirement for the homegarden through colorful ornamental landraces. Both in focus group discussion and interviews, the respondents indicated that surplus production and planting materials are sold in local markets and generate income for the household.

### Indigenous management and maintenance of enset diversity

Indigenous management and maintenance of on-farm enset landrace diversity can be deciphered by means of the three broad but overlapping indigenous approaches that farmers follow: (i) regular propagation and harvesting restrain; (ii) organized assemblage and arrangement of landraces in the homegarden; and (iii) landrace composition regulation in the homegarden. All the approaches the locals follow sustain on-farm landrace diversity.

#### Regular propagation and harvesting restrain

**Figure 6:**
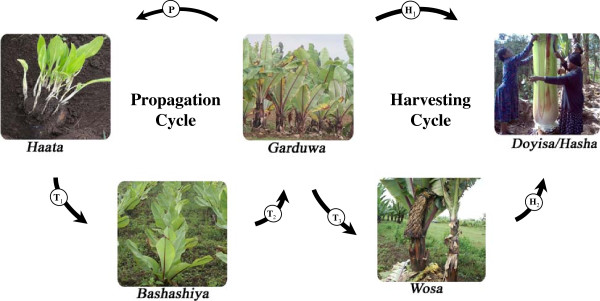
**A schematic representation of the enset cycle and management practices in Wolaita.** From *Garduwa* stage plants corms are selected for propagation (P) and corms buried for one year produce multiple suckers (*Haata*). *Haata* are transplanted (T1) and maintained for one year to give raise to *Bashashiya*, which after transplanting (T2) produce a new *Garduwa*. From these *Garduwa*, selected corms give rise to a new propagation cycle and the others are transplanted (T3) and kept as definitive establishment (*Wosa*) until harvest for processing (H2), or could be harvested (H1), especially in food-shortage seasons for cooking corms (*Doyisa uutta*).

Regularly propagating mixture of landraces and a balanced harvest restrain are practices farmers in the study area employ to regulate enset cycle and to maintain on-farm landrace diversity. The enset cycle managed by each of the household under study, from propagation to harvesting, involves maintaining 4 different successions of age-classes of plants in homegarden viz: *Garduwa*, *Haata*, *Bashashiya*, and *Wosa*, which distinguish propagation and harvesting cycle loops (Figure 6). According to informants, the enset cycle starts with the propagation, which is from plants in the *Garduwa* stage. Suckers are initiated from the *Garduwa* by burring the corms in 1 m deep pit after tipping the central shoot and removing apical dominance. The multiple suckers, raising from the buried corm (locally known as *Haata*) kept underground for 1 year, are separated from the mother corm and replanted in well manured nurseries. Replanted *Haata* grow in the nursery for another year after which they are named *Bashashiya*. A successive transplanting produces a new *Garduwa* (three years old plants). In addition of being used as mother corm for propagation, plants at *Garduwa* stage may be harvested for consumption, especially in food shortage seasons, or are transplanted as the definitive establishment, where they stay until harvested for processing (*Wosa* stage). Propagation is a cultural practice carried out every year, from late December to early February, using a mixture of landraces: on average, the farming households we studied propagate 5–15 plants per year before the on-set of the rainy season. Farmers select a mixture of landraces for propagation. Multipurpose landraces of high food and non-food uses such as *Ala-genna, Gefetanuwa, Halla, Maziya, Mochiya, Shalakumiya,* and *Tuzuma* are the most frequently propagated landraces every year. Other landraces are propagated alternatively year after year for maintenance and their limited number of use-values. However, some old and unproductive landraces are intentionally not propagated and are purged out from the homegarden or replaced with other landraces. Propagation as a practice is, therefore, not only a way for multiplying landrace of interest in homegarden but also part of the strategy for maintaining a dynamics of landrace diversity in the enset homegardens.

Harvesting for consumption and other uses is the second loop that completes the enset cycle (Figure 6). Harvesting a mixture of landraces with a balanced harvest restrain, both for the cooking- and processing-type landraces, is also a farmers' strategy of maintaining on-farm landrace diversity. Harvesting pressure is regulated by means of farmers' traditional beliefs, such as *cegena* (on-and off-set season of the moon), which is a fear factor that forces farmers not to harvest during this season, in the belief that enset plantation and processed product will be diseased and destroyed. Such convictions made farmers to distinguish and maintain landraces harvested early (e.g. *Gefetanuwa, Arkiya, Lembuwa*) and late (e.g. *Maziya, Argama, Wanadiyia*, *Halla*) before and after the on/off-set of *cegena* in their homegarden. The tradition of multi-enset food recipes and many other material culture needs, guide harvesting of landraces in mixture and reduce harvesting pressure of any particular landrace.

#### Regulation of enset landrace composition in homegardens

Multiple landraces, at least 2, are maintained and managed in the enset homegardens of Wolaita. The landraces maintained vary according to age (*Haata, Bashashiya, Garduwa and Wosa*), sex ('male' and 'female') and multiple usage. Farmers regulate and improve landrace composition of their homegardens to meet agronomic (e.g. early and late maturing landraces), cooking qualities and taste attributes (e.g. 'male' and 'female' landraces), aesthetic requirements (e.g. colorful landraces) and many other cultural values and needs. Farmer-to-farmer informal networks of 'seed sucker' transfers ensure the regulation of landrace composition in homegardens. Local farmers' informal pathways of planting materials include procurements from local markets (mainly in urban areas), gifts from neighbors and kinship groups, exchange for other landraces and bringing as living mementos from travels to add to their homegardens. Farmers' experimentation to regulate and improve homegarden landrace composition through networks of planting material systems maintains on-farm enset diversity in a dynamic way, both over time and space.

#### Organized assemblage and arrangement of landraces

The enset homegardens of the Wolaita are not a mere collection of landraces, rather there are specific arrangements and placements for each landrace. Farmers assemble landraces in a specific order for different purposes. For example, medicinally important landraces are planted in unsightly corners in the enset plantation, for 'preserving healing powers', whereas colorful landraces are planted along side-lines for ornamentation. The organized assemblage of landraces helps enset farmers to easily communicate the landraces they own to fellow farmers, which in turn helps to exchange and maintain landraces. In the community, well organized landraces in homegardens indicate scenic and symbolic values for the household, which again motivate farmers to maintain and manage more diversity in their homegardens. Specific arrangement and organization of landraces in the homegarden is one of the ways the Wolaita employ to maintain and manage on-farm enset diversity (Table 10).

**Table 10:** Planting arrangements of enset landraces in homegardens of Wolaita, Southern Ethiopia

**Landrace uses/characteristics**	**Representative landraces**	**Planting site in the homegarden**	**Justification for selection of planting site**
Ornamental/Colorful landraces	*Agino*, *Kambata, Maziya*	Along side-lines of farms	For ornamentation of homegardens
Vigorously growing landraces	*Ala-genna, Godariya, Halla, Mochiya, Wanadiyia*	Homegarden fringes	As symbol of status
Landraces used for water fetching from pseudostems	*Lalukiya*	Along homegarden paths	For ease of water fetching from the pseudostem
Medicinally important landraces*	*Argama, Gefetanuwa, Lochingia*	Unsightly corners inside the plantation	For preserving healing powers of landraces

*Maintaining medicinal landraces in unsightly corners of enset homegardens is less frequently practiced in Wolaita area at the present.

## Discussions

Enset cultivation is an integral part of rural livelihood for many ethno-linguistic communities in Southern Ethiopia, including the Wolaita [11]. This study documented IKS of enset intra-specific diversity, in order to identify the community practices and socio-cultural processes that drive on-farm maintenance of enset diversity in the Wolaita area. Data collected from key informants and 270 households residing in 12 *Kebeles*, selected through a stratified random sampling procedure from three agro-ecologies in Wolaita, confirmed the availability of a great wealth of IKS on enset agro-biodiversity in the studied area. The enset crop and its farming has enormous nutritional, socio-cultural, medicinal, environmental and economic values in the area, and if promoted it could highly contribute to sustainable food security and poverty reduction in rural areas.

### Status and importance of enset cultivation

According to our results, enset is cultivated by every farming household in Wolaita and it is the crop of choice for the community. In the agricultural system of the studied area, enset is the major perennial-stand permanent crop and most important food security source. Ages-old customary cultivation of enset in the area, which does not involve the use of chemical inputs [6,26], portrays an example of a small-scale, low-external-input and organic farming systems. According to farmers' perception, bacterial wilt disease, *wodhdh'ua*, (*Xanthomonas campestris* pv. *musacearum*), recurrent drought, demographic (e.g. increasing population size) changes and associated land shortages are threatening enset agriculture. This agrees with recent reports on the vulnerability of agricultural systems and agro-biodiversity in Southern Ethiopia [12,13,24].

### Intra-specific enset diversity and management

Our data confirms that farmers in the area of our study maintain the highest intra-specific diversity of enset than any other crop in their homegarden; the recorded richness (n = 67) and type of enset landraces in this study is comparable with other previous reports from different parts of Wolaita [11,24,34]. For example, of the recorded landraces in the present study, 43 were also reported in previous diversity study that documented 55 landraces from 1 *Kebele* (Areka) involving 105 households in the Boloso Sore *Woreda* of Wolaita Zone [11]. In another study that reported 20 landrace names from 4 *Kebeles* involving 156 respondents in Ofa *Woreda* of Wolaita Zone [23], 17 landraces were also found in our study. Twenty-four of the recorded landraces in our study were represented in the national enset *ex situ* AARC collection, where 37 enset landraces from Wolaita and many others from different enset growing ethno-linguistic communities of Southern Ethiopia are maintained (Figure 3-A). Although a direct comparison of landrace richness recorded in the present study with previous reports is not possible because of differences in the studied areas, number of households interviewed, sample size, year of study and the approaches followed, the higher number of landraces recorded in this study might be related to the stratification procedure applied and the relatively bigger sample size. However methodological differences between studies in spatial, temporal and sampling aspects can influence the outcome of on-farm landrace inventory, because communities in different areas maintain landrace suited to their socio-economic, environmental and cultural needs both over time and geographic scale [35].

A dynamic on-farm management of enset landraces, similar to what we documented here, was reported for Sidama, Gurage and Ari ethno-linguistic community in Southern Ethiopia [36-38]. Farmers value landrace diversity and exercise *de facto* conservation for their livelihood needs; during our focus group discussions and individual interviews farmers strongly expressed the desire and willingness to maintain more diversity in their homegarden. Amongst other reasons, farmers in our study area maintain a wide range of enset diversity and invest resources to sustain its cultivation with limited external supports and interventions as an insurance mechanisms against unexpected crop failures due to biotic and abiotic causes. The dynamic management followed by farmers we described here and methodological differences among the studies we reviewed, imply and call for continuous documentation and monitoring of landraces by applying a uniform and comparable procedures both over time and geographical scale for characterization and conservation of enset.

### Folk biosystematics and conservation perspectives

Folk biosystematics, i.e. identification, naming and classification of enset landraces, is an integral part of farmers decision of maintenance, management and exchange of landraces in the study area. A similar trend of using folk biosystematics was reported for enset in Sidama [34] and for sorghum (*Sorghum bicolor* (L.) Moench) in Eastern Ethiopia [39]. In view of this, farmers' given enset landrace names, perceived to represent distinct 'genotypes', have been used as farmers' diversity unit (FDU) for estimating the extent and distribution of enset diversity as well as *ex situ* collection expeditions in Southern Ethiopia, including Wolaita [24,36,40,41]. In the present study, FDU in Wolaita recognized 67 enset vernacular names (Table 3). Folk identification and naming of the recorded landraces was primarily based on agro-morphological descriptors of the plants (Table 4, 5). Folk descriptors used in our study area were comparable with descriptors reported from other enset growing areas, such as Sidama and Ari in Southern Ethiopia, where farmers also use mostly morphological features and use-value of enset landraces for identification [35,38,42]. Landrace names after plant characteristics, its perceived origin, various uses and culinary attributes are quite descriptive of specific landrace features (Table 5). However, for the majority of the reported landraces, names and their implied meanings were not explained by the local communities in the studied areas. Similar unexplained folk names of intra-specific farmers' varieties of sorghum in Ethiopia [39] and rice in Laos [43] were reported. Although identically named landraces as in our study were also reported from other ethno-linguistic groups [33,40], we did not attempt to directly compare landraces from other ethno-linguistic communities. In fact it is difficult to draw reliable conclusions on the identity of the landrace because of language differences. Nonetheless, exchange of enset planting material among ethno-linguistic groups could explain such 'borrowed' landrace names between ethno-linguistic groups.

For a crop such as enset that lacks a well established descriptor list and a formal taxonomic system [44,45], farmers' descriptors and folk biosystematics could be used as the basis for further investigations. Although farmers' diversity unit of enset gives a useful first approximation of the extent and distribution of enset diversity, studies complementing the extent to which farmer-named landraces are distinct agro-morphologically, biochemically and molecularly is highly needed. Amongst other benefits, a combination of folk and formal characterization (e.g. molecular) studies are crucial for minimizing synonym duplication and associated costs in *ex situ* collections that were reported for enset [46].

### Enset culture and use continuity consideration

Since time immemorial, the Wolaita have been using enset to meet their livelihood needs [6]. The community is interested in enset largely for its symbolic value as a cultural identity crop, its extensive use in material culture and culinary qualities as an indigenous staple. From the 10 types of enset dishes known in Wolaita, 8 can be prepared only from enset products and 2 (*Shendera, Eretta*) can be prepared from other crops like barley and maize with difference in texture and quality. The deep-rooted culture of using enset in daily lives from birth to death ceremonies and festive is an important bio-cultural heritage of the Wolaita as in other enset growing areas of Southern Ethiopia [6,23]. However, as reported by previous studies on the vulnerability of enset agricultural system in Southern Ethiopia, including Wolaita [11-13], farmer and key informant interviews in our study also indicated concerns about the continuity of the enset culture in the area due to the socio-cultural changes such as less appreciation and knowledge by the young and demographic changes such as increasing population and associated land shortages.

Strengthening local institutions and farmers leadership were demonstrated to support community efforts in conservation of agrobiodiversity in many indigenous communities [17,47]. Similarly, consideration of enset bio-cultural resource continuity needs to build on participatory community approaches and mobilization of both the young and the elder groups. Transmission of the knowledge system from one generation to the next should be facilitated through informal learning within family, relatives (keen-relationships) and neighborhoods. Organizing hands-on diversity management and enset cooking lessons from the more knowledgeable elder to the younger in community centers, in farmers' field school programs, might sustain this process. In addition, the integration of this knowledge system into the zonal/provincial education system can create opportunity for youth to learn and ensure the continuity of enset culture.

## Conclusion

This study provided a holistic view of the Wolaita enset culture. A great wealth of indigenous knowledge on the management and utilization of enset agro-biodiversity held by local communities in the Wolaita area was documented. Utilitarian and cultural reasons are the underlying drivers for on-farm maintenance and conservation of diverse enset landraces by the farmers. Complex and overlapping folk biosystematics and management practices underpin the enset cultivation. The documented information is crucial for developing complementary *in situ* and *ex situ* conservation approaches, but also for the promotion of a community based on-farm management of enset diversity in the area. The results presented here strongly reaffirm the potential of community based conservation efforts such as undertaken by the Wolaita in Southern Ethiopia for conservation of agrobiodiversity and imply the need to record and uncover the wealth of IKS of farming communities for sustainable conservation of biocultural diversity.

## Endnote

Landrace names and local terms were written in *Wolaitato Donaa* (the Wolaita Language).

## Abbreviations

CBD: The Convention on Biological Diversity; CSA: Central Statistical Agency, Ethiopia; FAO: Food and Agricultural Organization of the United Nations; FDU: Farmers’ diversity unit; IKS: Indigenous knowledge system; SNNPR: Southern People's, Nations and Nationalities Region.

## Competing interests

The authors declare that they have no competing interests.

## Authors' contributions

TMO carried out the field work and drafted the manuscript. BT conceived the study, followed up the field work and drafted the manuscript. MC participated in designing of the study and proof reading of the manuscript. MEP conceived the study, followed up the field work and coordinated the project. All authors read and approved the final manuscript.
